# Optical Rheology of Porcine Sclera by Birefringence Imaging

**DOI:** 10.1371/journal.pone.0044026

**Published:** 2012-09-06

**Authors:** Masahiro Yamanari, Kotaro Ishii, Shinichi Fukuda, Yiheng Lim, Lian Duan, Shuichi Makita, Masahiro Miura, Tetsuro Oshika, Yoshiaki Yasuno

**Affiliations:** 1 Computational Optics Group in the University of Tsukuba, Tsukuba, Japan; 2 Department of Ophthalmology, Institute of Clinical Medicine, University of Tsukuba, Tsukuba, Japan; 3 Department of Ophthalmology, Tokyo Medical University Ibaraki Medical Center, Ami, Japan; 4 Computational Optics and Ophthalmology Group, Tsukuba, Japan; Duke University, United States of America

## Abstract

**Purpose:**

To investigate a relationship between birefringence and elasticity of porcine sclera *ex vivo* using polarization-sensitive optical coherence tomography (PS-OCT).

**Methods:**

Elastic parameters and birefringence of 19 porcine eyes were measured. Four pieces of scleral strips which were parallel to the limbus, with a width of 4 mm, were dissected from the optic nerve head to the temporal side of each porcine eye. Birefringence of the sclera was measured with a prototype PS-OCT. The strain and force were measured with a uniaxial material tester as the sample was stretched with a speed of 1.8 mm/min after preconditioning. A derivative of the exponentially-fitted stress-strain curve at 0% strain was extracted as the tangent modulus. Power of exponential stress-strain function was also extracted from the fitting. To consider a net stiffness of sclera, structural stiffness was calculated as a product of tangent modulus and thickness. Correlations between birefringence and these elastic parameters were examined.

**Results:**

Statistically significant correlations between birefringence and all of the elastic parameters were found at 2 central positions. Structural stiffness and power of exponential stress-strain function were correlated with birefringence at the position near the optic nerve head. No correlation was found at the position near the equator.

**Conclusions:**

The evidence of correlations between birefringence and elasticity of sclera tested uniaxially was shown for the first time. This work may become a basis for *in vivo* measurement of scleral biomechanics using PS-OCT.

## Introduction

The elasticity of the collagen matrix in many types of tissues supports the biomechanical properties of organs. Particularly, the biomechanics of the eyeball has an important role in maintaining the round shape of the eye and protecting fragile structures of the retina. Although it has been known that the sclera of the eye has a significant role in the development and progress of myopia and glaucoma, [1], [2] it has proven difficult to measure scleral biomechanics noninvasively. Here, we demonstrate that scleral elasticity is associated with its birefringence, which can be measured using polarization-sensitive optical coherence tomography (PS-OCT). [3] We analyze the birefringence and elasticity of porcine sclera, and show correlations among these parameters. This technology for optical rheology is potentially applicable to the *in vivo* assessment of biomechanical properties of fibrous connective tissues, and may be a useful tool for investigating the progression of tissue remodeling.

It has been known that the sclera of myopic and glaucomatous eyes have different ultrastructural and viscoelastic properties from normal eyes. In myopic eyes, increased creep [4] and small collagen fibril diameters have been reported. [5] Remodeling and viscoelastic changes of the sclera are important features of high myopia, [1] which increase a risk of macular degeneration. In eyes with glaucoma, which is the second leading cause of blindness in the world, [6] the mechanical properties of the sclera have been considered to affect individual tolerance to intraocular pressure-related damage to the retinal nerve fibers [2]. Both increased stiffness and hypercompliance of the sclera have been reported in eyes with glaucoma. [7]–[12] Despite the importance of the scleral biomechanics for ocular diseases, it has been investigated only with *ex vivo* experiments and computer simulations because of the difficulty of obtaining *in vivo* measurements.

Although ultrasound elastography can measure the elasticity of a sample along the axial direction to the probe, [13] it cannot measure elasticity in the tangential direction, to which connective tissue often has organized orientation, e.g., of blood vessels, dermis and sclera. Elasticity of the collagen matrix in tissues is known to be influenced by the microstructure of collagen fibrils [14], which are finer than the resolution of optical microscopy. Polarized light microscopy (PLM) has been used to quantify such bundled fibers *in situ*, [15], [16] because the birefringence of the bundled fibers increases linearly with the number of fibers in the bundle [17]. However, it has been difficult to measure the birefringence of highly scattering tissues by PLM unless the sample is sliced. This limitation has prevented the measurement *in situ* of the elasticity of connective tissues.

PS-OCT is an interferometric method adapted to polarimetry, and can be used to measure the birefringence of such highly-scattering tissues. [3] High-resolution imaging with a few micrometer resolution and three-dimensional (3D) measurements in a few seconds have been demonstrated with recent advances of the technique. [18] It has been developed and applied to a wide variety of clinical investigations, e.g., ophthalmology, dermatology, cardiology and dentistry, etc. Although polarization-sensitive and birefringence images of various tissues have been demonstrated using PS-OCT, to the best of our knowledge, its potential for elasticity measurements of biological tissue has not yet been demonstrated. In this paper, we show for the first time the correlation between birefringence and elasticity of the sclera. This finding, although further extensive study is required, would imply future possibility of using PS-OCT for the assessment of tissue biomechanics.

## Methods

### Specimen preparation

**Figure 1:**
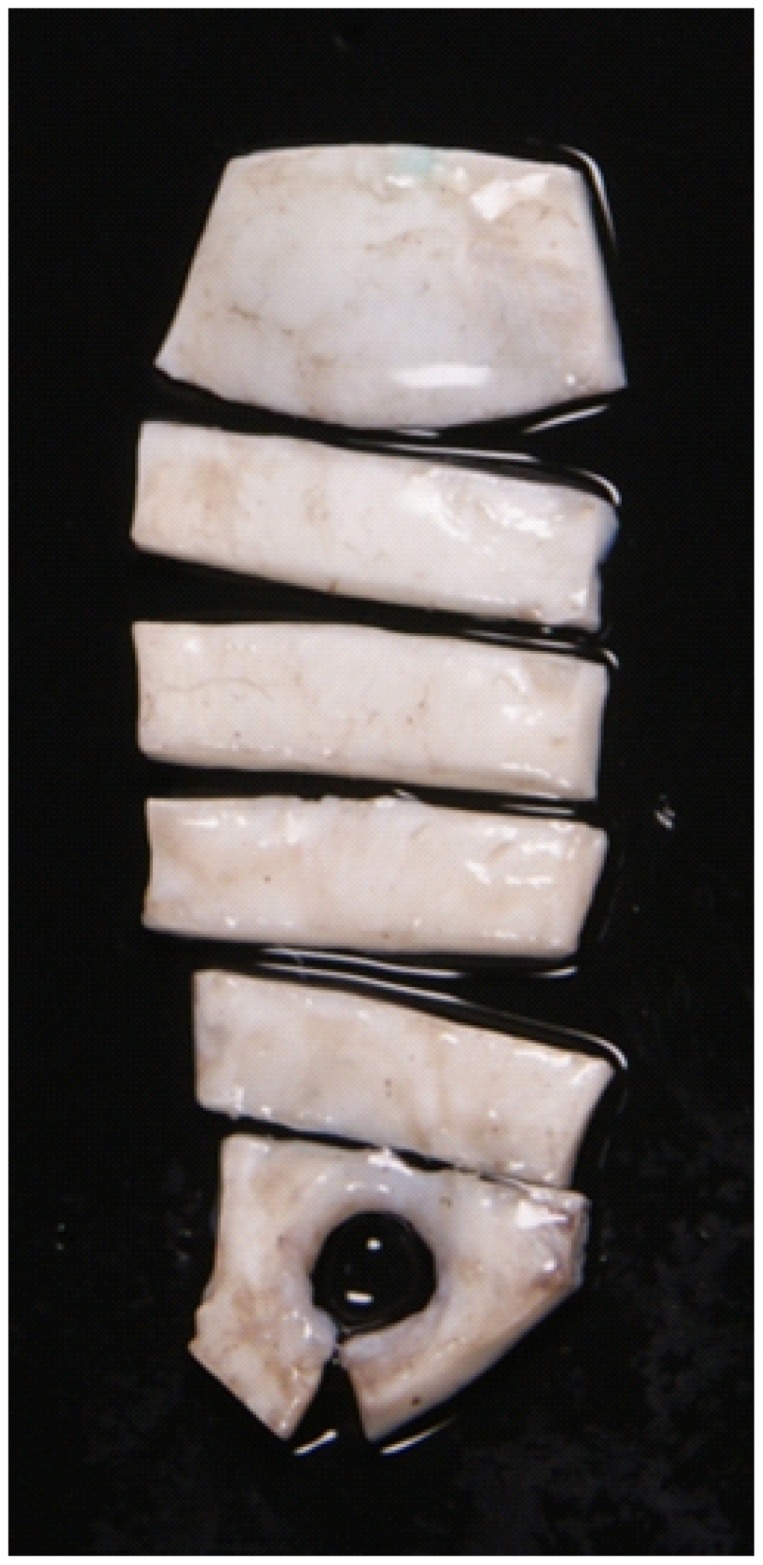
Porcine sclera dissected by scalpel blades. Strips in the lower and upper sides of the figure are peripapillary sclera and sclera near the limbus, respectively. The four strips of sclera between them were used for the measurement.

To investigate the correlation between the birefringence and elasticity of sclera, we measured these parameters of porcine sclera *ex vivo*. Nineteen porcine eyes with an age of 13 months were obtained from a local abattoir (Daimon Co., Ltd., Japan). The samples were measured within half a day of sacrifice. The sclera was dissected from the samples and cut with custom-made scalpel blades into four pieces (Fig. 1). Four pieces of scleral strips which were parallel to the limbus with a width of 4 mm were dissected from each porcine eye. The positions were set as distances from the optic nerve head to the temporal side to be 1–5 mm (Region-A), 5–9 mm (Region-B), 9–13 mm (Region-C), and 13–17 mm (Region-D). The region near the limbus was excluded because of non-uniform scleral thickness in this region. [19] During the dissection, the sample was cooled by an iced coolant.

**Figure 2:**
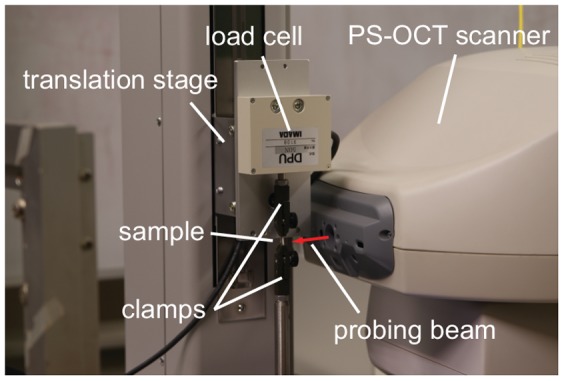
The experimental system setup for the birefringence and elasticity measurements.

The sample was fixed on a motorized translation stage (MX2-500N, Imada Co., Ltd., Japan) with two clamps at a distance of 5 mm (Fig. 2). During the experiment, saline was dropped on the tissue to keep its moisture.

### Measurement of cross-sectional area of sclera

**Figure 3:**
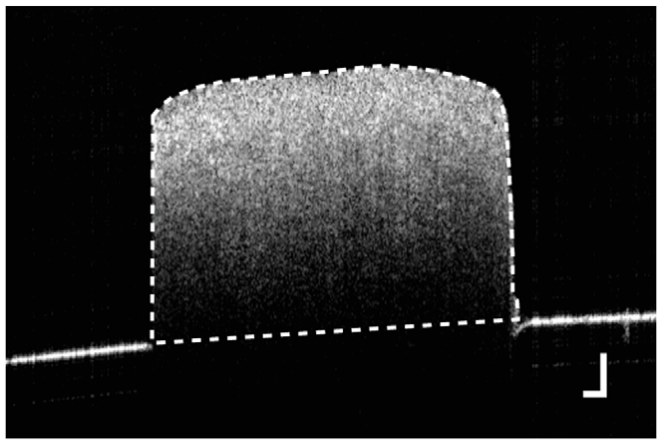
OCT image with a rubber plate for the measurement of the cross-sectional area. Since the edges of the rubber plate at left and right sides of the sclera correspond to the edges of a back surface of the sclera, the manually-segmented region indicated by the dashed curve shows the cross-sectional area of the sample in air. Scale bars show 250 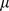
m.

First, a cross-sectional area of the sample, which was required for the calculation of elasticity, was measured by OCT. Since the porcine sclera is thick, the back surface of the sample was not imaged by OCT in all samples. In order to measure the cross-sectional area of the sample, a rubber plate was attached behind the sample during the OCT measurement for a surface topometry (Fig. 3). The area between the sample surface and the rubber plate at the center between the two clamps was defined as the cross-sectional area. It was measured by manual segmentation of the OCT image. Laterally-averaged thickness of the sample was calculated by taking the area divided by the width.

### Birefringence measurement

**Figure 4:**
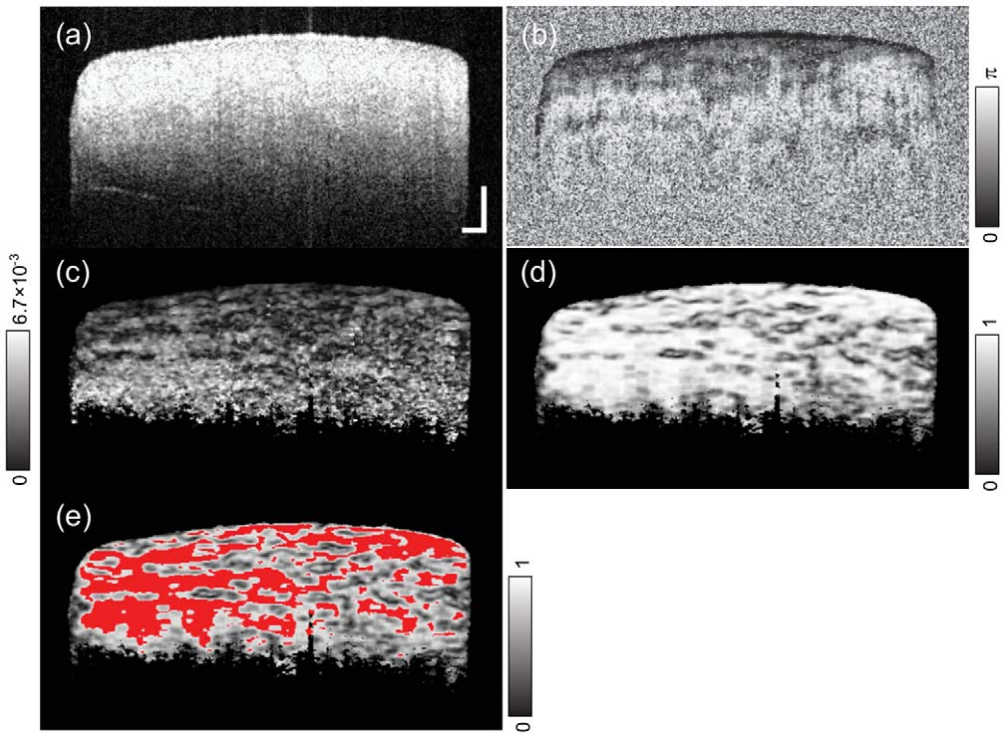
Representative B-scan images of the porcine sclera. OCT intensity image (a), phase retardation image (b) birefringence image (c), DOAU images (d) and (e) are shown. In figure (e), red color indicates the region above the threshold of 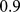 DOAU. Scale bars show 250 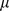
m.

Successively, OCT data of the sclera were acquired with a prototype PS-OCT developed in our laboratory. [20] The center wavelength of the light is 1.31 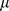
m. The axial and lateral resolutions of the system are 9.2 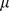
m and 20.5 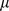
m, respectively. The outer surface of the sclera was set to face the scanner of the PS-OCT. A-scans of 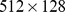 were acquired in the scanning range of 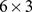 mm^2^ on the sample. A representative OCT intensity image at the center of the volumetric scan is shown in Fig. 4 (a). Since the collagen bundles are densely packed in sclera, it was difficult to distinguish the structure of collagen bundles and speckle. Smaller collagen structures, e.g., collagen fibrils, could not also be resolved with the axial resolution of OCT, which was 9.2 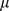
m in tissue.

To calculate birefringence, we developed a processing algorithm that is suitable for this study. From complex OCT data, 3D Jones matrices of the sample were calculated. Jones matrices were moving averaged with a kernel size of 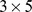 pixels (axial
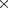
lateral). [20] Local round-trip Jones matrices were calculated with a pixel separation of 8 pixels (49 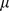
m). [21] Local birefringence was calculated using Lu-Chipman decomposition. [22] The obtained birefringence was corrected by a Monte Carlo-based mean estimator [23] and data obtained from pixels possessing an effective signal-to-noise ratio [21] of greater than 10 dB were utilized for subsequent analysis. Figure 4 (c) shows the birefringence image of the sclera. Birefringence is shown as the difference between the indices of refraction, which becomes a unitless value.

In Fig. 4 (c), birefringence was found not to be uniform in the sample, as expected from the anatomy of the sclera. [24] Because of the characteristic of our calculation method, the measured birefringence will have an artifact at the boundary of tissue domains which have different optic axes. To resolve this issue, the degree of optic axis uniformity (DOAU) was defined using Stokes parameters of eigenvectors of the Jones matrices as 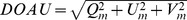, where 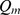, 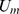, and 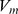 are Stokes parameters of axis orientations averaged in a kernel size of 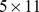 pixels (axial
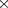
lateral, 31
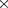
129 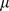
m^2^) centered at 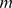-th pixel of the B-scan image. The eigenvectors were calculated by applying matrix diagonalization to the local round-trip Jones matrices. Figure 4 (d) shows DOAU image of sclera. Zero indicates a completely random orientation, and unity indicates a completely uniform orientation in the local regions of the kernel size. In contrast to the degree of polarization uniformity, which is a similar quantity to DOAU, [25] DOAU does not represent a state of polarized light, but can detect regions of nonuniform optic axes in the sample, which have unreliable local birefringence. Only the birefringence values obtained from pixels greater than DOAU of 0.9, which region was indicated in Fig. 4 (e), were used. Mean birefringence is calculated for each volume of the sample.

### Measurement of stress-strain response of the sclera

After the birefringence measurement, we measured the strain-stress response of the sample, and calculated elastic parameters, namely, tangent modulus, structural stiffness, and power of exponential stress-strain function.

**Figure 5:**
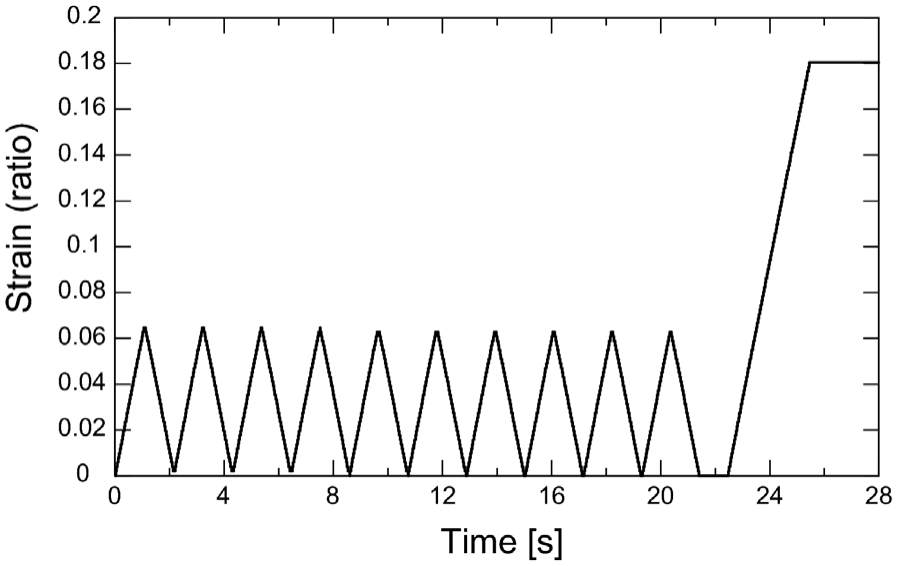
The applied strain to the sample.

The operation of the motorized translation stage is shown in Fig. 5. Pre-stress of 0.04 N was applied to take up any slack of the sample. The sample was extended by a motorized translation stage. Preconditioning was applied with ten cycles of loading and unloading from 0–6.5% strain with a speed of 1.8 mm/min. After 10 s of preconditioning, the sample was extended from 0–18% strain with the same speed. The strain and force were measured with a uniaxial material tester as the sample was stretched with a speed of 1.8 mm/min. This speed was sufficiently slow to apply the concept of pseudo-elasticity. [26]

### Calculation of elastic parameters

**Figure 6:**
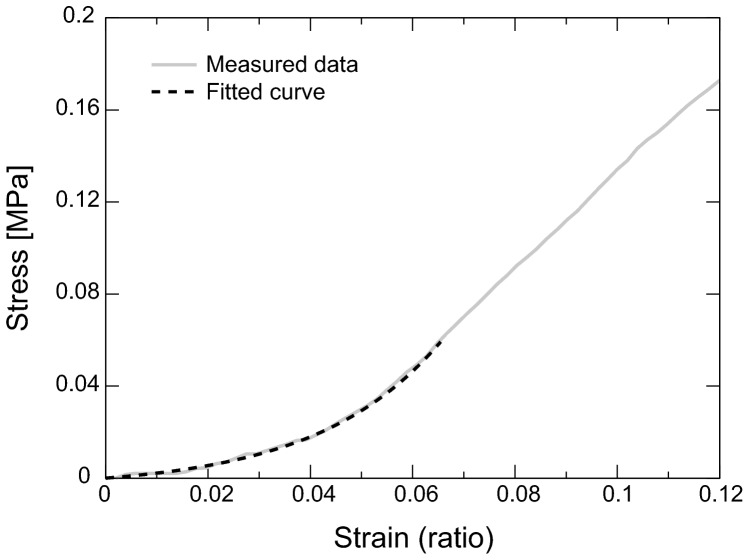
The measured strain-stress data and fitted curve from 0–6.5% strain.

The stress-strain curve was fitted with an exponential model, [27] 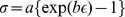, where 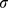 is tensile stress, which is the force applied to the sample divided by the initial cross-sectional area of the sample, and 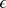 is the strain, namely, 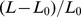, where 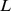 and 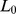 are the length of the sample at a certain time and the initial length of the sample, respectively. A strain range from 0–6.5% was used for the fitting. Since this strain range is the same with the range of the preconditioning, the sample is regarded as pseudo-elastic. Fig. 6 shows a representative stress-strain curve of the sclera.

Tangent modulus at 0% strain, 
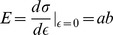
, was used for the analysis. To consider a net stiffness of sclera, structural stiffness was calculated as a product of tangent modulus and thickness. [28] Power of exponential stress-strain function, which was equivalent to the slope between tangent modulus and stress, 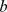, was also extracted from the fitting. [27]

### Statistical analysis of birefringence and elastic parameters

Two-sided tests using Pearson's product-moment correlation coefficient were applied to the birefringence and elastic parameters. For the statistical calculation, statistical computing language, R ver. 2.12.2 was used.

## Results

**Figure 7:**
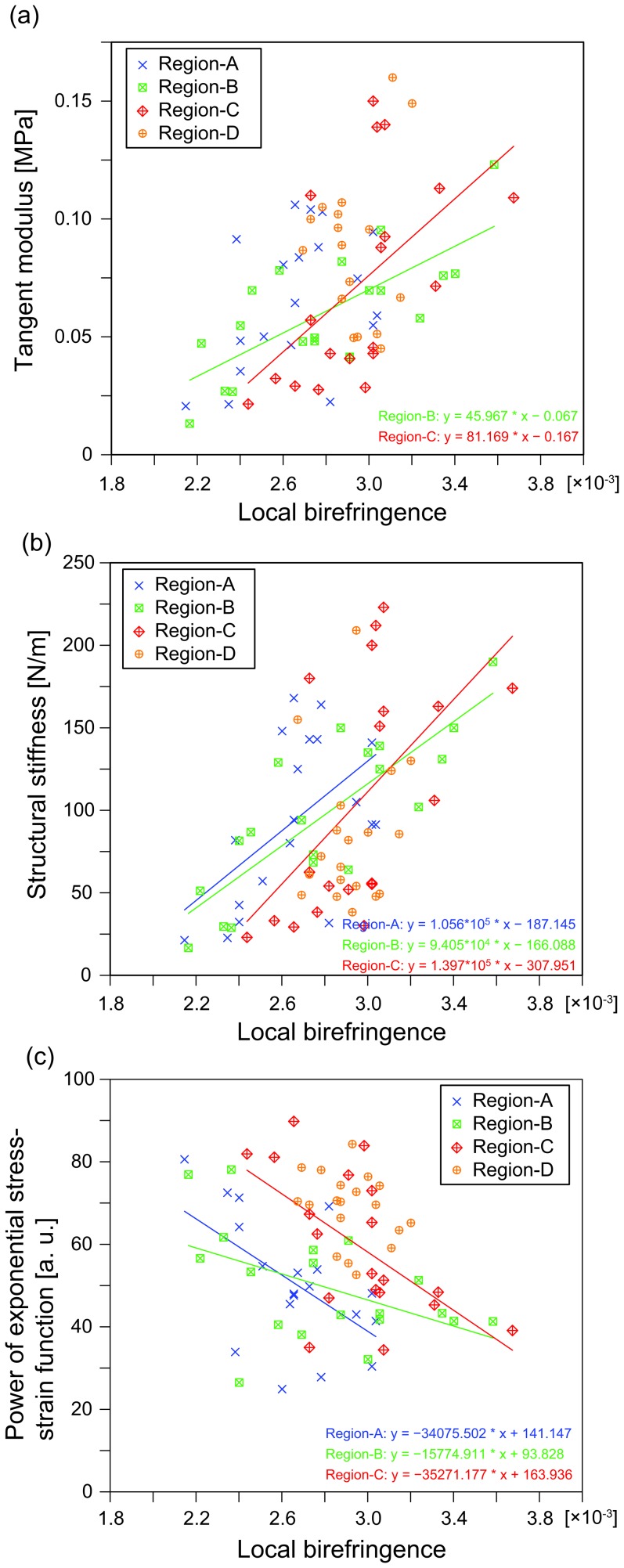
Plots of birefringence and tangent modulus (a), structural stiffness (b), and power of exponential stress-strain function (c) are shown. Linear regression lines are shown for the data that had statistically significant correlation in Table 1.

Figures 7 (a), (b) and (c) show data plots of the elastic parameters and local birefringence. Each plot represent each sample of sclera. Table 1 shows the correlation coefficients between birefringence and elastic parameters. As shown in Table 1, a positive correlation was found between birefringence and tangent modulus at the positions of Region-B and Region-C. Structural stiffness and power of exponential stress-strain function were also correlated with birefringence at the positions of Regions-A, B, and C. All elastic parameters at position D did not have statistically significant correlation with birefringence.

**Table 1:** Correlation coefficients (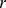) and 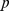-values between birefringence and elastic parameters at each position.

Positions		tangent modulus	structural stiffness	power of exponential stress-strain function
A	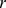	0.4092	0.5389*	−0.5463*
	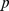	0.0819	0.0173	0.0155
B	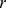	0.7402***	0.8242***	−0.4809*
	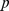	0.0003	1.43 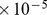	0.0371
C	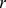	0.5429*	0.5630*	−0.5825**
	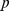	0.0163	0.0121	0.0089
D	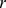	−0.1738	0.1222	−0.2821
	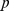	0.4766	0.6182	0.2419

Statistically significant correlations are marked with *, **, and *** for 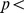
0.05, 0.01, and 0.001, respectively.

## Discussion

Tangent modulus and structural stiffness of the sclera were correlated with birefringence at several positions as shown in Table 1. These positive correlations can be understood as the highly organized collagen fiber had higher stiffness. In collagen fiber, each bundle is orientated with varying degrees of alignment, depending on the positions. [29]–[32] Since the direction of the applied uniaxial tension was parallel to the major orientation of the collagen fiber in the peripapillary and posterior sclera, namely, circumferential direction around the optic nerve head, [29], [30], [33] the elasticity measured at Regions-A, B, and C in our experiment would roughly represent the longitudinal elasticity of the collagen fiber. It is expected that elasticity along the perpendicular direction to the collagen fiber is lower than that of the longitudinal direction, depending on the degree of alignment of the collagen fibers. [29]–[32] We note that the above discussion is based on the experimental results of different samples in references but not of our samples, and the orientation and alignment of collagen fibers in sclera has not been fully understood. It would be helpful to perform further experiment with histology in future for investigating more details about the relationship between the birefringence and ultrastructure of the sclera. In addition, strain to another direction, such as an anteroposterior direction, would be worth to be investigated, since the deformation or remodeling of sclera along the anteroposterior direction may be related to the development of high myopia.

Power of exponential stress-strain function was correlated with birefringence at the positions of Regions-A, B, and C as shown in Table 1. Since it is known that more highly organized fibrous tissue has higher birefringence, [34], [35] this result might indicate that less organized tissue was highly stiffened during the extension. The nonlinear response of the sclera can be explained by the crimped and interwoven structure of the sclera. The sclera with less organized fiber might soon reach a linear response region of strained fiber. [28]

The reason why all elastic parameters at position D did not have any correlation with birefringence is unknown. We speculate that the sample size may be too large compared to the local variability of elasticity at this region, or that the contents of the sclera at this region might be different from others to support the connection to the extraocular muscle.

Since, in the current study, the samples were prepared at the same age and from a single local abattoir, the porcine eyes were likely to have limited variability, and hence linear regression was successfully applied to the relationships between birefringence and elastic parameters. However, the relationship could be nonlinear if uncontrolled subjects with a wider variety, e.g., age and diseases, were involved. In future, a theoretical analysis of form birefringence [36] would help in the development of a more rigorous mathematical model of the relationship. [17], [37]

Although we applied uniaxial tension to the sclera, the tension in the physiological condition, which is mainly caused by intraocular pressure, could be 3D and mainly biaxial along tangential directions to the ocular globe. To consider how the sclera resists the intraocular pressure, the orientation of collagen fiber in sclera would be an important factor. Scleral collagen fibers have different orientations depending on the areas of the eye. [33] Multilayered lamellae of the sclera has varying thickness and a crimped interwoven structure. [24], [38] Regional differences of scleral elasticity [27], [31] have implied the regional differences of such microstructure of the sclera. Deformation and remodeling of the sclera might be influenced by these and the 3D geometry of the sclera. In future, these factors will provide further understanding of the scleral biomechanics.

Although it is difficult to directly compare our results of stress-strain response of sclera with previous studies because of the different protocols of stress-strain measurements and different samples, we can say that our results showed lower tangent moduli than previous studies, because smaller strain (0%) was used to obtain tangent moduli in our experiment. [27], [39] In our study, we characterized the elastic property of porcine sclera by applying the preconditioning cycles and exponential fitting up to 6.5% strain. This strain is higher than the strain exhibited physiologically to the sclera *in vivo*, which would be on the order of 1%. [40] It also resulted in lower tangent moduli by the preconditioning cycles. In addition, since scleral stiffness increases with age [41], young porcine sclera used in our experiment might be more compliant than adult sclera. The above factors would partly explain the differences from other studies.

In this study, we used the prestress of 0.04 N in the experiment and the tangent modulus at 0% strain for the analysis, which are similar to *in vivo* condition of the eye. Although the condition of load testing does not exactly emulate *in vivo* strain, the measured elasticity might be interchangeable to the elasticity of sclera at low intraocular pressure *in vivo*.

Using magnetic resonance elastography, a measurement of an *ex vivo* bovine globe was reported. [42] Elastography measures tissue response to compressive vibration that is almost parallel to the imaging probe using a sonic transducer. However, since the loading direction and frequency are different from physiological stress to the sclera, its efficacy has not been shown for scleral measurements. In contrast, since the birefringence measured in this study is associated with scleral elasticity along the longitudinal direction, our approach would have a closer relationship with physiological stress to the sclera. In addition, PS-OCT measurement can be applied to *in vivo* eyes without any contact. This may suggest the possibility of making *in vivo* measurements of mechanical stiffness of the human eye, though further evidence would be required to show the potential of PS-OCT.

In this study, we focused on static property of scleral birefringence rather than its dynamic response during the extension, because the former would be important for noninvasive and noncontact *in vivo* measurement of the scleral property in future. However, the investigation of dynamic birefringence change of sclera during its extension would also be important. This is because such study would provide us an insight, for example, about dynamic scleral response to acute IOP increase, which has been considered as one of potential causes of retinal nerve fiber damage. [2], [28] It would be therefore our future work.

There would still be several challenges to measure biomechanical properties of the eye *in vivo* using PS-OCT in future. The correlation coefficients shown in Table 1 varied depending on the positions and elastic parameters. Since the choice of position in the eye and elastic parameter would influence the uncertainty of the elasticity estimation, it would be important to find the optimal position and optimal elastic parameters to be measured to have high association with the elasticity of sclera and progress of the diseases. For example, peripapillary sclera, which was not measured in this study, would be interesting to be included in the future studies, because it is known to be important for the deformation of the optic nerve head. [28] Finding the optimal parameter of the elasticity measurement, e.g., load testing orientation and the strain to calculate the tangent modulus, would also be important to interpret the measured birefringence as a certain elastic parameter that best represents the birefringence. Another issue of future *in vivo* studies would be a difficulty in direct elasticity measurement of *in vivo* sclera. It limits the designs of studies which assess the relationship between the elasticity and the birefringence of *in vivo* sclera. As a compromise, to evaluate the utility of PS-OCT as an *in vivo* assessment tool of diseases associated with scleral biomechanics, one of potential approaches would be to perform *in vivo* evaluation of the relationship between the birefringence of sclera and measurable biometric parameters, e.g., dioptric power, axial eye length, intraocular pressure, cup-to-disc ratio of the optic nerve head, and thickness of the retinal nerve fiber. The *ex vivo* experiments of the scleral elasticity would complement this possible *in vivo* study.

There were several limitations in our study. First, although it is known that the fibril diameter of the sclera depends on the depth, [1] we averaged birefringence at all depths. Depth-dependence of birefringence is the focus of a future study. Second, the sample was measured at room temperature controlled from 21–23 deg. The elastic properties of the sclera may be different at body temperature. Although it could cause a drift of elasticity, it would not significantly influence our analysis in this study. Third, as we mentioned, although we applied unidirectional stress to the sample in this experiment, the physiological stress to the sclera is 3D in general. Elasticity measurements with biaxial loading on the ocular globe have been reported, and showed stiffer response of the sclera than the results using uniaxial strain. [43] Such differences from the physiological condition should be carefully considered for *in vivo* measurements in future. Fourth, porcine sclera was used in this study. Although it is known that porcine and human sclerae have similar properties, [19], [44] it still remains to be demonstrated that the results shown here extend to human sclera.

Although the biomechanics of collagen gel have been investigated by PLM, [45], [46] none have demonstrated the biomechanics of dense connective tissue *in situ* with birefringence measurements. In this paper, the evidence of correlations between birefringence and elasticity of the sclera tested uniaxially was shown for the first time. Since birefringence of the sclera can be measured by PS-OCT in both the anterior and posterior segments of the *in vivo* human eye, [20], [47] optical rheology by PS-OCT may be able to estimate elasticity of the sclera at the major parts of the eyeball *in vivo*. It may also enable us to investigate the influence of scleral biomechanics to progressing myopia and glaucoma, and may help us monitor stiffening of the sclera by photochemical cross-linking of collagen for active control of the biomechanical property. [39] In addition to the ophthalmic application, this technology is possibly useful for studies of cross-linking of aged collagen, [48] cardiovascular imaging, where stiffness of blood vessel is important, and dermatology, where quantification of dermal elasticity is difficult.
